# Novel Specific Metallo-β-Lactamase Inhibitor ANT2681 Restores Meropenem Activity to Clinically Effective Levels against NDM-Positive *Enterobacterales*

**DOI:** 10.1128/AAC.00203-21

**Published:** 2021-05-18

**Authors:** Magdalena Zalacain, Clarisse Lozano, Agustina Llanos, Nicolas Sprynski, Thomas Valmont, Cyntia De Piano, David Davies, Simon Leiris, Carole Sable, Adeline Ledoux, Ian Morrissey, Marc Lemonnier, Martin Everett

**Affiliations:** a Antabio SAS, Labège, France; b IHMA Europe Sàrl, Monthey, Switzerland

**Keywords:** ANT2681, NDM, carbapenem-resistant *Enterobacterales*

## Abstract

The global dissemination of metallo-β-lactamase (MBL)-producing carbapenem-resistant *Enterobacterales* (CRE) is a serious public health concern. Specifically, NDM (New Delhi MBL) has been a major cause of carbapenem therapy failures in recent years, particularly as effective treatments for serine-β-lactamase (SBL)-producing *Enterobacterales* are now commercially available. Since the NDM gene is carried on promiscuous plasmids encoding multiple additional resistance determinants, a large proportion of NDM-CREs are also resistant to many commonly used antibiotics, resulting in limited and suboptimal treatment options. ANT2681 is a specific, competitive inhibitor of MBLs with potent activity against NDM enzymes, progressing to clinical development in combination with meropenem (MEM). Susceptibility studies have been performed with MEM-ANT2681 against 1,687 MBL-positive *Enterobacterales*, including 1,108 NDM-CRE. The addition of ANT2681 at 8 μg/ml reduced the MEM MIC_50_/MIC_90_ from >32/>32 μg/ml to 0.25/8 μg/ml. Moreover, the combination of 8 μg/ml of both MEM and ANT2681 inhibited 74.9% of the Verona integron-encoded MBL (VIM)-positive and 85.7% of the imipenem hydrolyzing β-lactamase (IMP)-positive *Enterobacterales* tested. The antibacterial activity of MEM-ANT2681 against NDM-CRE compared very favorably to that of cefiderocol (FDC) and cefepime (FEP)-taniborbactam, which displayed MIC_90_ values of 8 μg/ml and 32 μg/ml, respectively, whereas aztreonam-avibactam (ATM-AVI) had a MIC_90_ of 0.5 μg/ml. Particularly striking was the activity of MEM-ANT2681 against NDM-positive Escherichia coli (MIC_90_ 1 μg/ml), in contrast to ATM-AVI (MIC_90_ 4 μg/ml), FDC (MIC_90_ >32 μg/ml), and FEP-taniborbactam (MIC_90_ >32 μg/ml), which were less effective due to the high incidence of resistant PBP3-insertion mutants. MEM-ANT2681 offers a potential new therapeutic option to treat serious infections caused by NDM-CRE.

## INTRODUCTION

Bacterial production of β-lactamase enzymes, the main mechanism of resistance to β-lactam antibiotics in Gram-negative bacteria (1), has been increasing and evolving concomitantly with the introduction of new antibacterial agents from this chemical class. Marketing of third-generation cephalosporins and aztreonam in the 1980s was followed by the appearance of extended-spectrum β-lactamases (ESBLs) (2, 3), which made carbapenems the treatment of choice for serious infections caused by ESBL-positive organisms (4). However, new β-lactamases with activity against carbapenems started to emerge. Of these, Klebsiella pneumoniae carbapenemase (KPC) and oxacillinase (OXA-type) serine-β-lactamases (SBL), and New Delhi metallo-β-lactamase (NDM) have spread rapidly worldwide (5). Given the scarcity of novel antibiotics with activity against Gram-negative pathogens, carbapenem resistance threatens the last line of therapy for multidrug resistant hospital-associated infections and is a cause of serious clinical and economic concern (6, 7).

An alternative to the development of novel β-lactam antibiotics refractory to these newer enzymes is the use of β-lactamase inhibitors to restore the activity of those agents which are already available. This approach has yielded excellent ESBL and SBL inhibitors, such as avibactam, vaborbactam, and relebactam, but there are no specific metallo-β-lactamase (MBL) inhibitors in the market at the time of writing (recently reviewed in references 1, 8–11).

Serine-carbapenemases have been, and still are, the main global cause of carbapenem resistance, but NDM has become a significant cause of therapy failure in recent years in all regions. Although NDM was only reported for the first time in 2008, it already accounted for 10% of the carbapenemase genes among carbapenem-resistant *Enterobacterales* (CRE) collected in 2014 to 2016 as part of the SENTRY antimicrobial surveillance program, concomitant with a decrease in the two other major MBLs, namely, VIM (Verona integron-encoded MBL), from 2.4% to 1.9%, and IMP (imipenem hydrolyzing β-lactamase), from 1.9% to 0.4%, during the same time period (5). NDM has been detected in all continents but is most prevalent in the Indian subcontinent, the Balkan states, Nigeria, and Kenya (12).

ANT2681 is a specific, competitive small molecule inhibitor of MBLs (*K_i_* of 100 nM, 680 nM, and 6.3 μM versus VIM-1, VIM-2, and IMP-1, respectively; unpublished data) with particularly potent activity against NDM-1 (*K_i_*, 40 nM) (13, 14) and *in vivo* efficacy against NDM-positive CRE in murine thigh models of infection (15), which is being progressed to clinical development.

As part of the clinical studies performed with the meropenem (MEM)-vaborbactam combination (Vabomere), it has been shown that a MEM dosing regimen of 2 g infused intravenously (i.v.) over 3 h administered every 8 h is generally safe and well tolerated (16). As this dosing regimen achieves >90% probability of target attainment for MEM MICs ≤ 8 μg/ml (17, 18), we propose to evaluate it in combination with ANT2681.

Here, we describe the susceptibility studies performed to determine both the *in vitro* concentration necessary to potentiate MEM’s antibacterial activity to MICs ≤ 8 μg/ml and the effectiveness of this combination against a panel of MBL-positive CRE clinical isolates collected worldwide between 2015 and 2018.

## RESULTS

### MBL-positive isolates in a 2015 to 2018 global collection of *Enterobacterales* clinical isolates.

**FIG 1:**
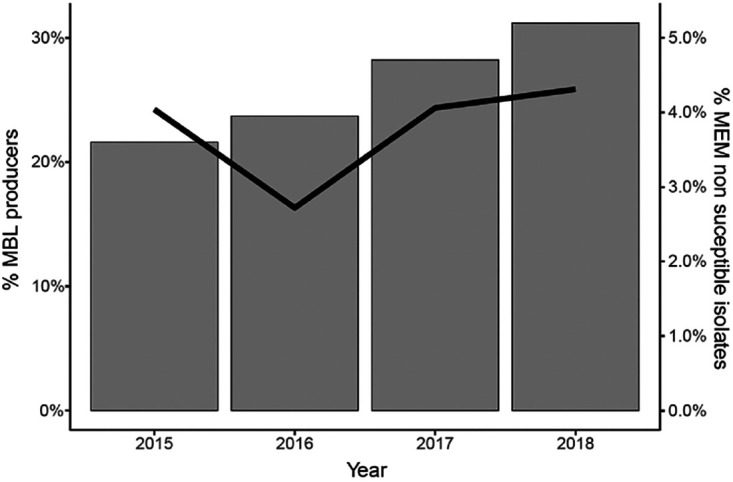
Percentage of MBL-positive isolates (histogram) within MEM-NS *Enterobacterales* (line) collected globally from 2015 to 2018.

**FIG 2:**
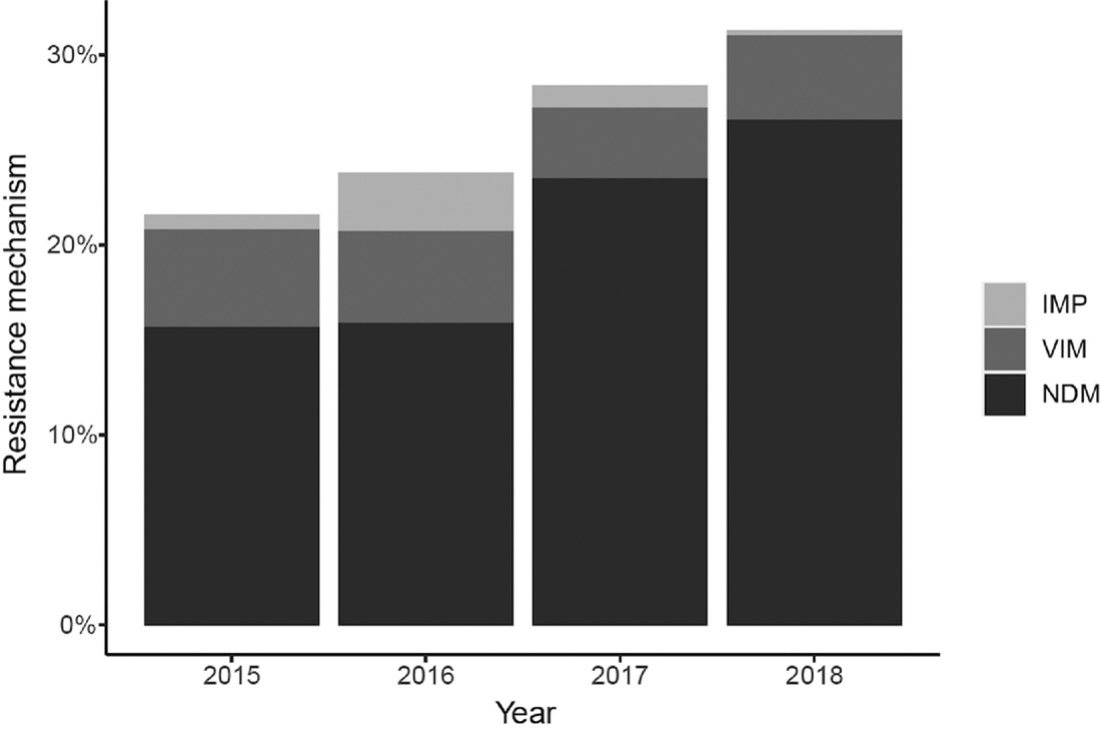
Percentage of isolates with NDM, VIM, and IMP resistance mechanism within MBL-positive MEM-NS *Enterobacterales* collected from 2015 to 2018.

A total of 218,521 *Enterobacterales* isolates were collected by IHMA from 2015 to 2018. Most of the isolates were collected in Europe (*n* = 94,132; 43.1%), followed by North America (*n* = 37,603; 17.2%), Asia/Pacific (*n* = 34,436; 15.8%), Latin America (*n* = 32,302; 14.8%), and Middle East/Africa (*n* = 20,048; 9.2%). Overall, 3.8% of isolates were nonsusceptible to MEM (MIC of ≥2 μg/ml). Approximately 75% of these (*n* = 6,072 isolates) were molecularly characterized for the presence of class A, B, and D carbapenemases. Although similar proportions of MEM nonsusceptible (MEM-NS) isolates were obtained over the 4-year period, 4%, 2.7%, 4%, and 4.3%, respectively (Fig. 1), a clear increase in the percentage of MBL-positive isolates could be observed within the MEM-NS population, from 21.6% in 2015 to 31.2% in 2018 (Fig. 1). This was driven by a statistically significant rise in NDM-positive *Enterobacterales*, which accounted for 15.7% of MBL-positive isolates in 2015 and for 26.6% in 2018 (*P* < 0.001), as well as the slight decrease in the percentage of the two other major MBLs, IMP (from 0.8% to 0.5%) and VIM (from 5.1% to 4.4%), over the same period (Fig. 2).

The antibacterial activity of MEM and MEM-ANT2681 was tested each year over a period of 4 years against all MBL-positive MEM-NS isolates collected from 2015 to 2018 as part of IHMA’s global surveillance study (total *n* = 1,687), with relevant comparator agents included against the 2017 and 2018 collections. Most of these MBL-positive isolates (94.1%) had ESBLs and some (*n* = 274; 16.2%) also carried a serine carbapenemase, with OXA-48-like being the predominant type (*n* = 250; 91.2%). By far the most common MBL+SBL combination was NDM+OXA (*n* = 233; 85.0%), with the rest distributed as follows: NDM+KPC (*n* = 12), VIM+OXA (*n* = 17), VIM+KPC (*n* = 11), and IMP+KPC (*n* = 1).

### Restoration of MEM antibacterial activity with different ANT2681 concentrations.

*In vitro* susceptibility testing against a small set of Gram-negative and Gram-positive organisms showed that ANT2681 had no intrinsic antibacterial activity (data not shown) and, therefore, its only role in the combination is to restore the activity of MEM to clinically efficacious levels.

Susceptibility testing of all 360 MBL-producing *Enterobacterales* (not carrying KPC or OXA) collected in 2016 was performed with MEM and increasing concentrations of ANT2681 in order to determine the level of ANT2681 necessary to restore MEM activity (MIC_90_ ≤ 8 μg/ml). The data showed that ANT2681 concentrations of ≥8 μg/ml were required to reduce the MEM MIC_90_ from >64 μg/ml to ≤8 μg/ml for all MBL-positive *Enterobacterales* and from >64 μg/ml to ≤4 μg/ml for NDM-producing CRE (Table 1). Further increasing the ANT2681 concentration provided little benefit and, therefore, ANT2681 was fixed at 8 μg/ml in the subsequent *in vitro* susceptibility studies.

**TABLE 1:** *In vitro* susceptibility of MEM with increasing concentrations of ANT2681 against MBL^+^ and NDM^+^ *Enterobacterales*

Antibacterial agent^*a*^	MIC (μg/ml)			
	MIC_50_	MIC_90_	MIN	MAX
MBL-*Enterobacterales* (*n* = 360) no OXA or KPC				
MEM	64	>64	2	>64
MEM-ANT2681 (4 μg/ml)	2	16	0.06	>64
MEM-ANT2681 (8 μg/ml)	1	8	0.03	>64
MEM-ANT2681 (16 μg/ml)	0.5	8	0.03	>64
MEM-ANT2681 (32 μg/ml)	0.25	4	0.03	>64
NDM-*Enterobacterales* (*n* = 247) no OXA or KPC				
MEM	64	>64	8	>64
MEM-ANT2681 (4 μg/ml)	1	16	0.06	>64
MEM-ANT2681 (8 μg/ml)	0.5	4	0.03	>64
MEM-ANT2681 (16 μg/ml)	0.25	4	0.03	64
MEM-ANT2681 (32 μg/ml)	0.25	2	0.03	64

a MEM, meropenem; NDM, New Delhi metallo-β-lactamase; MBL, metallo-β-lactamase; OXA, oxacillinase; KPC, K. pneumoniae carbapenemase.

### Antibacterial activity of MEM and MEM-ANT2681 (8 μg/ml) against different subsets of organisms.

Susceptibility studies were performed against 1,687 MBL-positive *Enterobacterales* and the results analyzed with regard to the presence of specific carbapenemases. The data confirmed that ANT2681 significantly potentiates MEM activity against a broad range of clinical isolates producing NDM as the sole carbapenemase (*n* = 1,108), reducing MEM MIC_90_ from >32 μg/ml to 8 μg/ml (Table 2). ANT2681 also restored MEM activity against the five isolates carrying NDM plus VIM, but it was less active against VIM-positive isolates (*n* = 251) and inactive versus IMP-positive organisms (*n* = 49) (Table 2). As expected, the effectiveness of the combination was substantially reduced if a serine-carbapenemase was also present in the organism. A slight MEM-potentiating effect was observed against *Enterobacterales* that carried NDM plus either KPC or OXA (*n* = 245), although not enough to be clinically useful.

**TABLE 2:** Number of 2015–2018 MBL^+^ *Enterobacterales* clinical isolates and cumulative % inhibited at specific MEM and MEM-ANT2681 (8 μg/ml) concentrations

Organism subset/drug^*a*^	No. of isolates and cumulative % inhibited at MIC (mg/liter) of:											
	Parameter	≤0.06	0.125	0.25	0.5	1	2	4	8^*b*^	16	32	>32
All MBL-positive (plus OXA/KPC) (*n* = 1,687)												
MEM	*n*	14	65	122	175	215	1096
	Cum%	0.8	4.7	**11.9**	22.3	35.0	100
MEM-ANT2681	*n*	120	230	278	219	156	103	120	102	95	171	93
	Cum%	7.1	20.7	37.2	50.2	59.5	65.6	72.7	**78.7**	84.4	94.5	100
All MBL-positive only (*n* = 1,413)												
MEM	*n*	14	64	113	154	186	882
	Cum%	1.0	5.5	**13.5**	24.4	37.6	100
MEM-ANT2681	*n*	118	229	275	212	143	90	107	86	55	47	51
	Cum%	8.4	24.6	44.0	59.0	69.1	75.5	83.1	**89.2**	93.1	96.4	100
All NDM only (*n* = 1,108)												
MEM	*n*	1	10	27	90	149	831
	Cum%	0.1	1.0	**3.4**	11.6	25.0	100
MEM-ANT2681	*n*	115	223	263	188	105	43	47	41	31	33	19
	Cum%	10.4	30.5	54.2	71.2	80.7	84.6	88.8	**92.5**	95.3	98.3	100
All NDM+OXA/KPC (*n* = 245)												
MEM	*n*	1	1	12	24	207
	Cum%	0.4	**0.8**	5.7	15.5	100
MEM-ANT2681	*n*	2	1	3	7	13	13	6	7	38	117	38
	Cum%	0.8	1.2	2.4	5.3	10.6	15.9	18.4	**21.2**	36.7	84.5	100
All VIM only (*n* = 251)												
MEM	*n*	7	44	61	59	33	47
	Cum%	2.8	20.3	**44.6**	68.1	81.3	100
MEM-ANT2681	*n*	3	6	11	19	34	40	47	28	21	13	29
	Cum%	1.2	3.6	8.0	15.5	29.1	45.0	63.7	**74.9**	83.3	88.4	100
NDM+VIM only (*n* = 5)												
MEM	*n*	1	2	2
	Cum%	20.0	60.0	100
MEM-ANT2681	*n*	1	4
	Cum%	20.0	100
All IMP only (*n* = 49)												
MEM	*n*	6	10	25	4	2	2
	Cum%	12.2	32.7	**83.7**	91.8	95.9	100
MEM-ANT2681	*n*	1	4	7	13	17	3	1	3
	Cum%	2.0	10.2	24.5	51.0	**85.7**	91.8	93.9	100

a MEM, meropenem; NDM, New Delhi metallo-β-lactamase; MBL, metallo-β-lactamase; IMP, imipenem hydrolyzing β-lactamase; VIM, Verona integron-encoded MBL; OXA, oxacillinase; KPC, K. pneumoniae carbapenemase.

b Highlighted in boldface are the % of strains inhibited by MEM at 8 μg/ml.

In these studies, the combination of MEM and ANT2681, both at 8 μg/ml, inhibited the growth of 78.7% of the entire collection of *Enterobacterales* tested and of 89.2% of strains that carried only an MBL carbapenemase. Specifically, it inhibited 92.5% of the NDM-CRE isolates, 74.9% of the VIM-positive, and 85.7% of the IMP-positive *Enterobacterales* (Table 2). This concentration of MEM-ANT2681 also inhibited growth of 21.2% of NDM-positive strains in which a KPC or OXA carbapenemase was also present (Table 2).

### Antibacterial activity of MEM and MEM-ANT2681 (8 μg/ml) against different NDM variants.

Most of the NDM-positive CREs evaluated in this study carried NDM-1 (77.3%), followed by NDM-5 (10.2%), and NDM-7 (4.7%). MEM-ANT2681 displayed MIC_90_ values of 8 μg/ml against NDM-1-positive isolates and MIC_90_ values of 1 μg/ml against CREs with the other two most common NDM variants (Table 3).

**TABLE 3:** Activity of MEM-ANT2681 against isolates with different NDM variants

NDM variant^*a*^	No.	Drug^*b*^	MIC (μg/ml)		
			MIC_50_	MIC_90_	Range
All	1,108	MEM	>32	>32	2 to >32
		MEM-ANT2681	0.25	8	≤0.06 to >32
NDM-1	857	MEM	>32	>32	2 to >32
		MEM-ANT2681	0.25	8	≤0.06 to >32
NDM-4	31	MEM	>32	>32	4 to >32
		MEM-ANT2681	0.5	2	≤0.06 to >32
NDM-5	113	MEM	>32	>32	8 to >32
		MEM-ANT2681	0.5	1	≤0.06 to 16
NDM-6	23	MEM	>32	>32	8 to >32
		MEM-ANT2681	0.5	1	≤0.06 to 4
NDM-7	52	MEM	>32	>32	8 to >32
		MEM-ANT2681	0.25	1	≤0.06 to >32
Other^*a*^	32	MEM	>32	>32	8 to >32
		MEM-ANT2681	1	32	≤0.06 to >32

a NDM, New Delhi metallo-β-lactamase. NDM-9 (*n* = 10), NDM-15 (*n* = 1), NDM-16 (*n* = 2), NDM-19 (*n* = 1), NDM-24 (*n* = 1), NDM-type (*n* = 17).

b MEM, meropenem.

Similar MIC_90_ values were obtained against isolates carrying NDM-4 (*n* = 31) and NDM-6 (*n* = 23), but a higher value was observed against the pool of organisms carrying all other NDM variants (Table 3). This is due to the fact that 9/10 of the NDM-9 isolates, representing a third of the strains in the pooled group, had MEM-ANT2681 MICs of ≥16 μg/ml. There is a high probability that these particular isolates are clonally related, as they were all identified as NDM-9/CTX-M-15/SHV-OSBL K. pneumoniae and all were collected in Guatemala, with 8/9 isolated in 2018 from the same hospital. The fact that MEM-ANT2681 displays a MIC of 0.25 μg/ml against the only non-Guatemalan NDM-9 K. pneumoniae isolate, collected in the Philippines, indicates that ANT2681 is active against this NDM variant and that some other determinant(s) accounts for the high MIC in the Guatemalan strains. This remains to be confirmed by additional studies.

### Comparative analysis of the antibacterial activity of MEM-ANT2681 and comparator antibiotics against NDM-CRE clinical isolates.

Several comparator agents, either marketed drugs or combinations in clinical development with activity against MBL-CREs, were also tested against one or both of the 2017 and 2018 NDM-CRE collections (Table 4). The activity of MEM-ANT2681 (MIC_90_, 8 μg/ml; 92.5% inhibited at this concentration) compared favorably to that of other antibiotics tested, such as cefepime (FEP)-taniborbactam (MIC_90_, 32 μg/ml), cefiderocol (FDC) (MIC_90_, 8 μg/ml; 84.3% susceptible), colistin (CST) (MIC_90_, >16 μg/ml), and eravacycline (ERV) (MIC_90_, 2 μg/ml; 52.2% susceptible) (Table 4). The only other combination with clear potential for clinical activity against NDM-CRE was aztreonam-avibactam (ATM-AVI) (MIC_90_, 0.5 μg/ml) (Table 4).

**TABLE 4:** *In vitro* susceptibility data for MEM, MEM-ANT2681, and comparator antibiotics against NDM-CRE isolates from 2017 and 2018 global collections

Isolates/antimicrobial agent^*d*^	Total no.	No. of isolates and cumulative % inhibited at MIC (mg/liter) of:												MIC_50_	MIC_90_	CLSI^*e*^	
		Parameter	≤0.06	0.125	0.25	0.5	1	2	4	8	16	32	>32			% Sus	% Res
2017 Global Collection^*a*^																	
All NDM-positive	213																
MEM		*n*	2	6	23	45	137
		Cum%	0.9	3.8	14.6	35.7	100	>32	>32	0	100
MEM-ANT2681		*n*	33	34	47	34	20	5	8	17	7	7	1
		Cum%	15.5	31.5	53.5	69.5	78.9	81.2	85.0	93.0	96.2	99.5	100	0.25	8	-	-
ATM-AVI		*n*	53	76	56	14	4	4	3	1	2
		Cum%	24.9	60.6	86.9	93.4	95.3	97.2	98.6	99.1	100	0.12	0.5	-	-
FEP-taniborbactam		*n*	1	3	16	58	44	29	12	11	22	17
		Cum%	0.5	1.9	9.4	36.6	57.3	70.9	76.5	81.7	92.0	100	2	32	-	-
NDM-K. pneumoniae	141																
MEM		*n*	1	2	9	30	99
		Cum%	0.7	2.1	8.5	29.8	100	>32	>32	0	100
MEM-ANT2681		*n*	9	25	37	27	12	4	4	13	5	4	1
		Cum%	6.4	24.1	50.4	69.5	78.0	80.9	83.7	92.9	96.5	99.3	100	0.25	8	-	-
ATM-AVI		*n*	21	60	49	10	1
		Cum%	14.9	57.4	92.2	99.3	100	0.12	0.25	-	-
FEP-taniborbactam		*n*	6	46	37	21	6	6	13	6
		Cum%	4.3	36.9	63.1	78.0	82.3	86.5	95.7	100	2	32	-	-
NDM-E. coli	32																
MEM		*n*	1	7	8	16
		Cum%	3.1	25.0	50.0	100	32	>32	0	100
MEM-ANT2681		*n*	16	5	1	6	3	1
		Cum%	50.0	65.6	68.8	87.5	96.9	100	≤0.06	1	-	-
ATM-AVI		*n*	13	6	2	2	4	2	1	2
		Cum%	40.6	59.4	65.6	71.9	84.4	90.6	93.8	100	0.12	4	-	-
FEP-taniborbactam		*n*	1	10	6	1	2	5	7
		Cum%	3.1	34.3	53.1	56.3	62.5	78.1	100	1	>32	-	-
NDM-E. cloacae	15																
MEM		*n*	1	1	5	8
		Cum%	6.7	13.3	46.7	100	>32	>32	0	100
MEM-ANT2681		*n*	2	2	5	5	1
		Cum%	13.3	26.7	60.0	93.3	100	0.25	1	-	-
ATM-AVI		*n*	1	5	7	1	1
		Cum%	6.7	40.0	86.7	93.3	100	0.25	0.5	-	-
FEP-taniborbactam		*n*	2	3	5	4	1
		Cum%	13.3	33.3	66.7	93.3	100	4	8	-	-
NDM-Other species	25																
MEM		*n*	3	6	2	14
		Cum%	12.0	36.0	44.0	100	>32	>32	0	99.2
MEM-ANT2681		*n*	6	2	4	1	4	4	1	3
		Cum%	24.0	32.0	48.0	52.0	68.0	84.0	88.0	100	0.5	32	-	-
ATM-AVI		*n*	18	5	1	1
		Cum%	72.0	92.0	96.0	100	≤0.06	0.12	-	-
FEP-taniborbactam		*n*	1	2	4	3	3	2	3	3	4
		Cum%	4.0	12.0	28.0	40.0	52.0	60.0	72.0	84.0	100	4	>32	-	-
2018 Global Collection^*b*^																	
All NDM-positive	452
MEM		*n*	1	8	14	30	48	351
		Cum%	0.2	2.0	5.1	11.7	22.4	100	>32	>32	0	99.8
MEM-ANT2681		*n*	48	117	113	77	33	12	9	10	12	15	6
		Cum%	10.6	36.5	61.5	78.5	85.8	88.5	90.5	92.7	95.4	98.7	100	0.25	4	-	-
FEP-taniborbactam		*n*	5	14	41	128	87	49	34	37	24	33
		Cum%	1.1	4.2	13.3	41.6	60.8	71.7	79.2	87.4	92.7	100	2	32	-	-
FDC		*n*	7	4	6	13	56	149	146	45	12	1	13
		Cum%	1.5	2.4	3.8	6.6	19.0	52.0	84.3	94.2	96.9	97.1	100	2	8	84.3	5.8
CST		*n*	66	284	13	4	5	9	19	52^*c*^
		Cum%	14.6	77.4	80.3	81.2	82.3	84.3	88.5	100^*c*^	0.5	>16	-	18.8
ERV		*n*	1	10	76	149	116	63	32	4	1
		Cum%	0.2	2.4	19.3	52.2	77.9	91.8	98.9	99.8	100	0.5	2	52.2	-
NDM-K. pneumoniae	275
MEM		*n*	2	4	4	19	246
		Cum%	0.7	2.2	3.6	10.6	100	>32	>32	0	100
MEM-ANT2681		*n*	13	76	75	47	16	7	3	8	11	13	6
		Cum%	4.7	32.4	59.6	76.7	82.6	85.1	86.2	89.1	93.1	97.8	100	0.25	16	-	-
FEP-taniborbactam		*n*	5	25	95	58	28	18	19	9	18
		Cum%	1.8	10.9	45.5	66.6	76.7	83.3	90.2	93.5	100	2	16	-	-
FDC		*n*	6	42	117	93	13	2	2
		Cum%	2.2	17.5	60.0	93.8	98.5	99.3	100	2	4	93.8	1.5
CST		*n*	23	200	10	2	5	8	17	10^*c*^
		Cum%	8.4	81.1	84.7	85.5	87.3	90.2	96.4	100^*c*^	0.5	8	-	14.5
ERV		*n*	1	40	99	84	33	15	2	1
		Cum%	0.4	14.9	50.9	81.5	93.5	98.9	99.6	100	0.5	2	50.9	-
NDM-E. coli	59
MEM		*n*	6	6	47
		Cum%	10.2	20.3	100	>32	>32	0	100
MEM-ANT2681		*n*	9	10	14	17	7	1	1
		Cum%	15.3	32.2	55.9	84.8	96.6	98.3	100	0.25	1	-	-
FEP-taniborbactam		*n*	1	3	4	4	1	6	15	13	12
		Cum%	1.7	6.8	13.6	20.3	22.0	32.2	57.6	79.7	100	16	>32	-	-
FDC		*n*	3	14	17	11	4	10
		Cum%	5.1	28.8	57.6	76.3	83.1	100	4	>32	57.6	23.7
CST		*n*	32	25	1	1
		Cum%	54.2	96.6	98.3	100	0.25	0.5	-	0
ERV		*n*	9	23	19	5	3
		Cum%	15.3	54.2	86.4	94.9	100	0.25	1	86.4	-
NDM-E. cloacae	58
MEM		*n*	1	6	5	13	33
		Cum%	1.7	12.1	20.7	43.1	100	>32	>32	0	100
MEM-ANT2681		*n*	9	10	15	5	10	4	4	1
		Cum%	15.5	32.8	58.6	67.2	84.5	91.4	98.3	100	0.25	2	-	-
FEP-taniborbactam		*n*	3	16	9	16	9	2	2	1
		Cum%	5.2	32.8	48.3	75.9	91.4	94.8	98.3	100	4	8	-	-
FDC		*n*	3	6	27	15	5	1	1
		Cum%	5.2	15.5	62.1	87.9	96.6	98.3	100	4	16	62.1	12.1
CST		*n*	10	43	1	1	1	2^*c*^
		Cum%	17.2	91.4	93.1	94.8	96.6	100^*c*^	0.5	0.5	-	6.9
ERV		*n*	9	23	12	7	7
		Cum%	15.5	55.2	75.9	87.9	100	0.5	4	55.2	-
NDM-Other species	60
MEM		*n*	1	5	4	15	10	25
		Cum%	1.7	10.0	16.7	41.7	58.3	100	32	>32	0	98.3
MEM-ANT2681		*n*	17	21	9	8	1	1	1	2
		Cum%	28.3	63.3	78.3	91.7	93.3	95.0	96.7	100	0.125	0.5	-	-
FEP-taniborbactam		*n*	5	8	10	13	16	4	1	1	2
		Cum%	8.3	21.7	38.3	60.0	86.7	93.3	95.0	96.7	100	1	4	-	-
FDC		*n*	7	4	6	7	8	12	9	6	1
		Cum%	11.7	18.3	28.3	40.0	53.3	73.3	88.3	98.3	100	1	8	88.3	1.7
CST		*n*	1	16	2	1	40^*c*^
		Cum%	1.7	28.3	31.7	33.3	100^*c*^	>16	>16	-	68.3
ERV		*n*	1	4	8	15	20	10	2
		Cum%	1.7	8.3	21.7	46.7	80.0	96.7	100	2	4	21.7	-

a 2017: C. freundii (*n* = 2), K. oxytoca (*n* = 1), P. mirabilis (*n* = 3), *P. rettgeri* (*n* = 10), P. stuartii (*n* = 8), and *R. ornithinolytica* (*n* = 1).

b 2018: C. freundii (*n =* 5), *E. asburiae* (*n =* 1), *Enterobacter* spp. (*n =* 2), K. aerogenes (*n =* 4), K. oxytoca (*n =* 9), M. morganii (*n =* 4), P. mirabilis (*n =* 6), *P. rettgeri* (*n =* 4), P. stuartii (*n =* 15), S. marcescens (*n =* 10).

c Number of isolates and cumulative % inhibited at MIC of >16 μg/ml.

d NDM, New Delhi metallo-β-lactamase; MEM, meropenem; ATM, aztreonem; AVI, avibactam; FEP, cefepime; FDC, cefiderocol; CST, colistin; ERV, eravacycline.

e CLSI susceptibilities as defined by the CLSI document M100-S30 (2020) have been used, except for eravacycline, where the breakpoints have been defined by FDA (2018). S breakpoints used: MEM, ≤1 μg/ml; FDC, ≤4 μg/ml; ERV, ≤0.5 μg/ml. R breakpoints used: MEM, ≥4 μg/ml; FDC, ≥16 μg/ml; CST, ≥4 μg/ml. The symbol “-” indicates not applicable.

All of the Enterobacter cloacae isolates, 99.0% of Escherichia coli, and 90.3% of K. pneumoniae strains from the 2017 and 2018 collections had MEM-ANT2681 MICs of ≤8 μg/ml (Table 4). FEP-taniborbactam had MIC_90_ values of 8, >32, and 32 μg/ml against these subsets of organisms, respectively, whereas MIC_90_ values for FDC were 16, >32, and 4 μg/ml, respectively, and ATM-AVI displayed MIC_90_ values of 0.5, 4, and 0.25 μg/ml, respectively. CST and ERV demonstrated variable activity against these three subsets, with MIC_90_ values ranging from 0.5 to 8 μg/ml for CST, and from 1 to 4 μg/ml for ERV (Table 4).

### Activity of MEM-ANT2681 versus NDM-E. coli isolates carrying an insertion in PBP3.

ATM-AVI, FDC, and FEP-taniborbactam displayed noticeably higher MIC_90_ values for NDM-E. coli isolates than for other NDM-positive species. It was speculated that this may be due to the presence of mutations in PBP3, the primary penicillin-binding protein target for several β-lactams, insertions in which have been reported to contribute to reduced susceptibility to ATM, FDC, and FEP (19–21). Hence, the *ftsI* gene (which encodes PBP3) was amplified and sequenced from 30 E. coli isolates that displayed ATM-AVI MICs ≥ 4 μg/ml (5 collected in 2017) or FDC MICs ≥ 8 μg/ml (25 collected in 2018). All these E. coli isolates also displayed FEP-taniborbactam MICs ≥ 16 μg/ml. Sequencing data revealed that all isolates had a four amino acid insertion at position 333 of the PBP3 protein (29 isolates having YRIN and 1 isolate having YRIK). Recently, Sadek et al. (21) have shown that the presence of this modified PBP3 is not sufficient to confer resistance to ATM-AVI and, in fact, all resistant isolates investigated in their study also carried CMY-42. CMY enzymes were also identified in the five E. coli that displayed ATM-AVI MICs ≥ 4 μg/ml. Although these PBP3 mutations do not affect activity of carbapenems, the presence of NDM in these particular isolates resulted in MEM MICs of ≥32 μg/ml. Addition of ANT2681 decreased MEM MICs against these E. coli isolates to between 0.06 and 4 μg/ml.

## DISCUSSION

Antimicrobial resistance is seriously compromising modern medicine and our way of life. Among antibiotic-resistant bacteria, CRE has been classified as a priority 1 pathogen by WHO (22) and as an urgent threat by the CDC (23), and there is a global consensus that novel therapies are needed to treat infections caused by these Gram-negative pathogens.

The first case of KPC-producing K. pneumoniae emerged in the US in 1996 and was reported in 2001 (24). Ten years later, this resistance mechanism had disseminated worldwide and had been detected in all clinically relevant *Enterobacterales* (25). Fortunately, by the time this carbapenemase emerged, the β-lactamase inhibition potential of the diazabicyclooctanes (DBO) and boronic acid derivatives had already been discovered (26, 27) and development of novel β-lactamase inhibitors with anti-KPC activity proceeded rapidly, resulting in the launch of ceftazidime-avibactam (2015), meropenem-vaborbactam (2017), and imipenem-relebactam (2019). Likewise, NDM, first reported in 2008 (28), had been detected in all continents within 6 years (29), and already accounted for 10% of CRE isolates collected worldwide in 2014 to 2016, as recorded by the SENTRY Antimicrobial Surveillance Program (5). Moreover, molecular characterization of the global 2015 to 2018 collection of MEM-NS *Enterobacterales* used in this study shows a clear increase in the percentage of MBL-positive isolates, from 21.6% in 2015 to 31.2% in 2018, driven primarily by a statistically significant rise in NDM-positive isolates, from 15.7% of the MBL-positive isolates in 2015 to 26.6% in 2018. However, in contrast to the situation when KPC first emerged, there were no preexisting MBL inhibitors in development and the difficulty in identifying novel and specific MBL inhibitors, together with the currently limited commercial potential in major pharmaceutical markets, has made progress in this area slow. Hence, safe and effective treatment options for serious infections caused by NDM-CRE are still required.

ANT2681 is the culmination of a chemistry program designed to deliver a potent and selective NDM inhibitor. This compound shows no P450 inhibition, hERG inhibition, mammalian cytotoxicity, or genotoxicity, is selective toward other metalloenzymes (14), and was well tolerated in safety toxicology/pharmacology studies at exposures well above those necessary for efficacy (15). As the area under the concentration curve from 0 to 24 h (AUC_0–24_) is the pharmacodynamic index driving ANT2681 efficacy (15) it should be feasible to administer an efficacious dose in combination with 2 g MEM, administered as a 3-h infusion every 8 h, which would provide coverage of CREs with MEM MICs of up to 8 μg/ml.

ANT2681 significantly potentiates MEM activity against a broad range of NDM-producing clinical isolates (including all NDM variants present in this study), reducing MEM MIC_50_/MIC_90_ from >32/>32 μg/ml to 0.25/8 μg/ml. In addition, the combination of MEM and ANT2681, both at 8 μg/ml, inhibited the growth of 74.9% of the VIM-positive and 85.7% of the IMP-positive *Enterobacterales* tested.

MEM-ANT2681 inhibits growth of 92.5% of NDM-CRE isolates at the anticipated susceptibility breakpoint (BP) of ≤8 μg/ml MEM and compares favorably to the activity of comparator drugs, such as FDC (84% susceptibility at the CLSI BP of ≤4 μg/ml) or FEP-taniborbactam, a combination being evaluated in phase 3 clinical trials (30) (78.3% susceptibility at the CLSI BP for FEP of ≤8 μg/ml). Of the additional comparator drugs tested (ATM-AVI, CST, and ERV), ATM-AVI was the only one that appears to have the clinical potential to treat NDM-CRE. While ATM is a monobactam and is not hydrolyzed by MBLs, MBL-positive isolates often carry ESBLs which do hydrolyze it (31). Since AVI is an effective inhibitor of ESBLs, the combination ATM-AVI could be an effective treatment option against MBL-producing CREs (32) and is currently undergoing phase 3 clinical trials. In the meantime, clinicians are already administering this combination by using two drugs already approved by the FDA, namely, ATM and CAZ-AVI (33–35). This practice provides a very clear indication of the shortness of drugs available to treat these deadly pathogens.

There are other combinations at different stages of clinical development with reported activity against NDM-positive CRE. Meropenem-nacubactam (36) and cefepime-zidebactam (37) rely on the direct antibacterial activity of the BLi for their effect against MBL-CRE. QPX7728 is a dual SBL and MBL inhibitor, reported to be developed as a partner to different β-lactam antibiotics, which shows a very good microbiological profile when combined with MEM (38).

Worryingly, several recent publications report the emergence of E. coli isolates harboring a four-amino-acid insertion in PBP3, either YRIN or YRIK after residue 333 (19-21), or TIPY after residue 344 (39), which contribute to the reduced susceptibility of these isolates to β-lactam antibiotics. These strains, which have evolved through mutation and recombination and have been selected by β-lactam use, have started to disseminate globally and seem to preferentially acquire carbapenemase genes (40); they represent a new emerging clinical threat, as most novel β-lactam and β-lactam/BLI combinations, including FDC, FEP-taniborbactam, and ATM-AVI, are not active against them. In fact, MIC_90_ values for these drugs against the subset of NDM-E. coli tested in this study were higher than those obtained against the entire collection of NDM-CRE, and molecular characterization of 30 of these isolates with reduced susceptibility confirmed the presence of the YRIN/YRIK insertion at position 333 of PBP3 in all cases. As the lack of MEM activity against these E. coli isolates is due to the presence of NDM, MEM-ANT2681 effectively restores the carbapenem activity, lowering MEM MICs to ≤4 μg/ml, and is thus a very promising therapeutic option for this important emerging threat.

In summary, ANT2681 restores MEM activity to clinically effective levels against NDM-CRE and, therefore, the MEM-ANT2681 combination represents a novel and potentially effective therapy to treat serious or life-threatening infections caused by these organisms, for which there are few treatment options that offer both efficacy and a favorable safety and tolerability profile. The availability of targeted narrow-spectrum drugs such as MEM-ANT2681 would support antibiotic stewardship efforts by helping to conserve broad-spectrum drugs for medical situations that require immediate empirical therapy or for which no other treatment option is available.

## MATERIALS AND METHODS

### Compounds.

The following antimicrobial agents (doubling dilution range) were tested in this study: MEM (0.004 to 32 μg/ml), MEM-ANT2681 (0.03/8 to 32/8 μg/ml), aztreonam-avibactam (ATM-AVI) (0.03/4 to 32/4 μg/ml), cefepime (FEP)-taniborbactam (0.03/4 to 32/4 μg/ml), cefiderocol (FDC) (0.03 to 32 μg/ml), colistin (CST) (0.12 to 16 μg/ml), and eravacycline (ERV) (0.015 to 32 μg/ml).

ANT2681-Na and taniborbactam were synthesized by GVK-Biosciences (Hyderabad, India). ATM was purchased from Biokemix (Leese, Germany). AVI was obtained from Biochempartner (Wuhan, China). FDC was purchased from Chem Scene (NJ, USA). ERV was obtained from MedChemTronica (Stockholm, Sweden). MEM, FEP, and CST were purchased from the U.S. Pharmacopeia (Rockville, MD).

### Bacterial strains.

*Enterobacterales* tested in this study (*n* = 1,687) were selected from the IHMA Europe Sàrl, (Monthey, Switzerland) 2015 to 2018 global surveillance studies frozen stock culture collection based on their MEM nonsusceptible phenotype (MEM MIC of ≥2 μg/ml) (41) and the presence of an MBL gene. All *Enterobacterales* were originally isolated from specimens of patients (one isolate per patient) with a documented intraabdominal, urinary tract, skin and soft tissue, lower respiratory tract, or bloodstream infection. The identities of all isolates were confirmed by IHMA using matrix-assisted laser desorption ionization–time of flight (MALDI-TOF) mass spectrometry (Bruker Daltonics, Billerica, MA).

The presence of the following clinically relevant β-lactamase genes had been evaluated by PCR as described previously (42): (i) genes encoding Ambler class A extended-spectrum β-lactamases (SHV-type, TEM-type, CTX-M-1-type, CTX-M-2-type, CTX-M-8-type, CTX-M-9-type, CTX-M-25-type, and GES-type); (ii) class A serine carbapenemases (KPC, GES, and IMI/NMC-A); (iii) class B metallo-β-lactamases (NDM, IMP, VIM, SPM, and GIM); (iv) class C plasmid-mediated AmpC-type β-lactamases (ACC, ACT, CMY, DHA, FOX, MIR, and MOX); and (v) class D serine carbapenemases (OXA-48-like). Genes encoding SHV, TEM, GES-type, KPC, OXA-48-like, NDM, IMP, and VIM β-lactamases were amplified, sequenced, and compared to the database maintained by the United States National Center for Biotechnology Information (www.ncbi.nlm.nih.gov) to determine enzyme subtypes. The *ftsI* gene (encoding PBP3) was also amplified by PCR and sequenced in 30 E. coli isolates with elevated FDC or ATM-AVI MICs.

These isolates were collected in Europe (*n* = 705), Asia/South Pacific (*n* = 395), Middle East/Africa (*n* = 339), Latin America (*n* = 228), and North America (*n* = 20) from 2015 to 2018 and included Citrobacter braakii (*n* = 2), Citrobacter farmeri (*n* = 3), Citrobacter freundii (*n* = 52), Citrobacter koseri (*n* = 3), *Citrobacter* spp. (*n* = 1), Enterobacter asburiae (*n* = 6), Enterobacter cloacae (*n* = 277), Enterobacter kobei (*n* = 3), *Enterobacter* spp. (*n* = 7), Escherichia coli (*n* = 169), Klebsiella aerogenes (*n* = 7), Klebsiella oxytoca (*n* = 31), Klebsiella pneumoniae (*n* = 992), Morganella morganii (*n* = 5), Proteus mirabilis (*n* = 23), Providencia rettgeri (*n* = 24), Providencia stuartii (*n* = 51), Raoultella ornithinolytica (*n* = 1), and Serratia marcescens (*n* = 30). No isolates from India or China were included in this study.

### Antimicrobial susceptibility testing.

Antimicrobial susceptibility testing of MEM-ANT2681 and comparator agents was performed by broth microdilution following Clinical and Laboratory Standards Institute (CLSI) guidelines using custom panels prepared by IHMA (41, 43) in cation-adjusted Mueller-Hinton broth (CAMHB) (Becton, Dickinson). Iron-depleted CAMHB was used to test FDC. The panels were incubated at 35°C for 16 to 20 h in ambient air before MIC endpoints were read visually. MIC values corresponded to the first well with no visible growth.

Quality control (QC) testing was performed each day of testing as specified by CLSI using the following isolates: E. coli ATCC 25922, Pseudomonas aeruginosa ATCC 27853, and K. pneumoniae ATCC 700603. MICs were interpreted using either CLSI or EUCAST (European Committee on Antimicrobial Susceptibility Testing) guidelines or both when available (41, 44).

MEM and MEM-ANT2681 were tested each year over a period of 4 years against all MBL-positive MEM-NS isolates collected by IHMA during the previous year as part of their global surveillance studies. These criteria were applied all years regardless of the comparators tested in each study. Comparator drugs were tested only one or 2 years, ATM-AVI (2017), FEP-taniborbactam (2017, 2018), and CST, FDC, and ERV (2018).

### Statistical analysis.

The chi-square test for trend with one binary variable and one ordered categorical variable was applied following the method described in reference 45. A *P* value of < 0.05 was considered statistically significant. Bar plots were generated using the R package “ggplot2” (46).
